# Expression of IL-21 and IL-33 in Intestinal Mucosa of Inflammatory Bowel Disease: An Immunohistochemical Study

**DOI:** 10.3390/diagnostics13132185

**Published:** 2023-06-27

**Authors:** Alexandros Toskas, Stefanos Milias, Georgios Delis, Soultana Meditskou, Antonia Sioga, Theodora Papamitsou

**Affiliations:** 1 Laboratory of Histology and Embryology, Medical School, Aristotle University of Thessaloniki, 54124 Thessaloniki, Greece; sefthym@auth.gr (S.M.); sioga@auth.gr (A.S.); thpapami@auth.gr (T.P.); 2 St Marks Hospital, Watford Rd, Harrow, London HA1 3UJ, UK; 3 Private Histopathology Laboratory, Ploutonos 27, 54655 Thessaloniki, Greece; smilias@yahoo.com; 4 Veterinary School, Aristotle University of Thessaloniki, 54124 Thessaloniki, Greece; delis@vet.auth.gr

**Keywords:** IL-21, IL-33, IBD, Crohn’s, UC, immunochemistry, biologics

## Abstract

Interleukins are considered to be potential therapeutic targets that can alter the prognosis and disease progression of IBD. IL-21 has proven to be involved in effector Th1, Th2 and Th17 responses. Similarly, IL-33, a newly identified cytokine, has been shown to control the Th1 effector response and the action of the colonic Tregs in animal models of colitis and patients with IBD. In this retrospective study, we have studied the expression of these interleukins, using immunohistochemistry, in 121 patients with moderate to severe IBD before and after treatment with biologics. The results were statistically processed using SPSS^TM^. Increased IL-21 expression was found in the UC and CD groups versus the controls. The IL-33 expression was found to be increased in the post-treatment UC and CD groups, suggesting a protective role of this interleukin against bowel inflammation. The IL-33 expression post-treatment was reversely correlated with the activity index score in CD patients, suggesting a better response to treatment in patients with higher IL-33 mucosa levels. This is the first immunohistochemical study of the expression of those interleukins in bowel mucosa before and after treatment with biologics. These data support a possibly promising future use of these interleukins as biomarkers of severe disease and response to treatment and as potential therapeutic targets for novel monoclonal antibodies.

## 1. Introduction

Interleukins have lately been considered as potential therapeutic targets in Inflammatory Bowel Disease (IBD). The imbalance of the immune response against the bowel flora is considered to be the main trigger for Crohn’s disease (CD) and ulcerative colitis (UC). CD has features of an exaggerated predominant T helper (Th) 1 cell response with production of interferon (IFN)-γ and interleukin-12 (IL-12), and UC mainly has a (Th) 2 response that is dominated by the production of IL-5 and IL-13. However, both conditions share the same end-stage effector pathways of tissue damage, featuring enhanced production of IL-21 [1].

Evidence suggests that IL-21 is associated with the development of IBD in humans. IL-21, a member of the IL-2 family of cytokines, is mainly expressed by CD4^+^ T helper cells, including Th1, Th2 and Th17 cells, and controls the immune process by (a) enhancing the clonal expansion of antigen-activated naive CD4^+^ and CD8^+^ T-cells, (b) inducing the expression of genes encoding IL-12R, IL-18R, IFN-γ, IL-2Rα and Th1-associated transcription factor T-bet in activated-memory T-cells, (c) promoting human Natural Killer (NK) cell maturation and activation, (d) regulating the differentiation and antibody production of B-cells, (e) stimulating stromal cells to produce tissue-degrading proteases and (f) enhancing the secretion of the T-cell chemoattractant macrophage inflammatory protein-3a via intestinal epithelial cells [2,3]. The results of different studies of IL-21 in the pathogenesis of IBD contradict each other. It has been shown that IL-21 can provoke colonic inflammation, and its neutralization may have therapeutic potential for patients with IBD [4]. Other studies have shown that IL-21/IL-21R(receptor) signaling may suppress intestinal inflammation in Dextran Sulphate Sodium (DSS)-induced colitis in mice through the suppression of Th1 and the activation of Th2 and Treg responses. Recombinant IL-21 administration improved DSS-induced colitis, suggesting that manipulation of the IL-21/IL-21R activity may have a role in the management of IBD [5].

IL-33 is a newly described member of the IL-1 family, expressed in a variety of non-haemopoietic cells, certain populations of inflammatory cells and epithelial cells of the intestinal mucosa. It seems to help maintain gut homeostasis [6,7,8,9]. It is suggested that IL-33 has a dual function: as a pro-inflammatory cytokine, mainly inducing Th2 cell response, and as a regulatory cytokine, alerting the immune system of danger or tissue damage when released from injured epithelial cells. IL-33 levels were increased in parasitic infections of the gastrointestinal tract and in active UC [10,11,12,13,14,15].

In this study, we aim to investigate the trends of the expression of IL-21 and IL-33 in IBD patients and healthy controls in regard to disease activity and before and after treatment in separate groups treated with biologics. This is a retrospective single-center study using immunohistochemistry. We also aim to investigate the role of those interleukins in IBD pathogenesis and the possible utilization of creating prognostic models to predict the response to treatment and disease behavior.

## 2. Materials and Methods

### 2.1. Data Collection

A detailed history of 121 patients with IBD was taken from the archives of the Gastroenterology Department of 424 General Military Hospital of Thessaloniki, between 1999 and 2017. The age, gender, disease extent, severity and treatment choice were recorded. Endoscopy reports before and after 12 months of treatment were retrieved. Intestinal biopsy samples were retrieved from the Histology and Histopathology Lab before and 1 year after treatment with biologics. A written consent was signed by the patients, and the study was approved by the Bioethics Committee of Aristotle University of Thessaloniki.

### 2.2. Study Groups

The initial sample was divided into 2 groups depending on the type of the IBD: (A) Crohn’s disease (CD) and (B) ulcerative colitis (UC). Those two groups were then divided into 3 subgroups based on the biologic treatment they had received: 1. Anti-TNFa (Tumour Necrosis Factor a) treatment (Adalimumab (ADA) and Infliximab (IFX); 2. Ustekinumab (USK); and 3. Vedolizumab (VDZ). The activity of the disease was measured before and 1 year after treatment using the Harvey-Bradshaw score for CD (HBI) and the Mayo score for UC (MS). Specimens from twenty non-IBD patients were used as controls.

### 2.3. Immunohistochemistry

The antibodies used were the IL 21 rabbit polyclonal (ab154767, ABCAM Ltd., Cambridge, UK) and the IL 33 monoclonal (clone 12B3C4, NOVUS Biologicals Ltd., Oxon, UK). The following process was repeated for each of the antibodies used.

From each patient, a paraffin block from the archives was obtained. Two sections of each block (4 μm in thickness) were cut and placed in unstained, positively charged slides. A positive control from a lymph node and a negative control from the pancreas were also cut and placed in the same glass. Each slide was deparaffinized in the incubation chamber (Thermo^TM^ Scientific, Waltham, MA, USA) at 60.5 °C for 45 min. The immunohistochemistry protocol was completed with a BOND polymer refined detection kit with the IHC protocol F. The slides were then stained in a fully automatic immunostainer Bond-MAX^TM^ (Leica^TM^ Biosystems, Nussloch, Germany) that completed the following steps: (a) blocking of peroxide for 5 min., (b) marker incubation for 15 min., (c) heat-induced epitope retrieval (HIER) ER1 for 30 min. (for both antibodies), (d) post-primary for 8 min., (e) polymer for 8 min., (f) mixed DAB for 10 min., and (g) hematoxylin for 5 min. At the end of each cycle, the slides were rehydrated in ascending alcohols (75°, 95°, 100°), cleared in two steps of Xylene and then covered with slide covers using mounting medium.

Microscopic evaluation was then performed using an optical Nikon^TM^ microscope, and photographs were taken using a Euromex^TM^ scientific camera attached to the microscope. The intensity of staining was evaluated as negative (−), weak (+), moderate (++) or strong (+++).

### 2.4. Statistical Analysis

The data collected were analyzed using descriptive and regression statistics. ANOVA was used to compare the intensity of staining between controls and IBD patients. SPSS^TM^ Version 29 was used for the analysis, and Microsoft Excel ^TM^ was used for the creation of charts and graphs.

For the purpose of the statistical analysis, the patients were grouped into 5 categories based on their age: 1. 21–30, 2. 31–40, 3. 41–50, 4. 51–60, and 5. >61.

## 3. Results

A total number of 72 patients with Crohn’s disease (57 male, 15 female) and 49 patients with ulcerative colitis (40 male, 9 female) were studied.

The minimum age for the Crohn’s disease patients was 21 years old, and the maximum age was 81 years old. The minimum age for ulcerative colitis patients was 21 years old with a maximum of 85 years old.

### 3.1. Duration of the Disease (Table 1)

The duration of disease in IBD patients was shown in Table 1.

**Table 1:** Duration of disease in IBD patients.

Disease	Duration	Frequency	Percent
UC	<3 years	11	22.4
	>3 years	38	77.6
	Total	49	100.0
CD	<3 years	8	11.1
	>3 years	64	88.9
	Total	72	100.0

### 3.2. IBD Phenotype

The patients were divided according to the disease phenotype using the Montreal Classification (E1-3) and S (1-3) based on the extent and the severity of the disease. The UC patients were evenly distributed regarding the severity of the disease, with most of them having left-sided colitis/pancolitis (Table 2). Most Crohn’s disease patients had ileocolonic disease (41.7%) that was non-stricturing or penetrating (65.3%). A total of 98.6 % of the Crohn’s disease patients included in this study were older than 17 years of age, which was expected, as the data were collected from an adult gastroenterology service. Only two patients with upper GI involvement (2.8% of the Crohn’s patients) were involved.

### 3.3. Activity of the Disease

#### 3.3.1. Mayo Score for Colitis

The UC patients (*N* = 49) were divided into 3 categories based on the Mayo score for colitis, with each part rated from 0 to 3, giving a total score of 0 to 12. A score of 3 to 5 points indicated mildly active disease, a score of 6 to 10 points indicated moderately active disease and a score of 11 to 12 points indicated severely active disease. The minimum Mayo score on presentation was 3, with a maximum score of 12. A total of 8 patients (16.3%) had mildly active colitis on presentation with a Mayo score of 3–5, 27 patients had a score between 6 and 10 (61.3%) and 11 patients had a Mayo score between 11 and 12 (22.4 %).

#### 3.3.2. Harvey-Bradshaw Index (H.B.I.) for Crohn’s Disease

The CD patients (*N* = 72) were divided into four categories based on the HBI presentation. A total of 5.6 % were considered to be in clinical remission with an HBI < 5. A total of 15.2% had mildly active CD, with an HBI between 5 and 7; 54.2% had moderately active CD, with an HBI between 8 and 16; and 25% had severely active CD with an HBI > 16 on presentation.

### 3.4. Treatment Choices of IBD Patients

Most of the UC patients (61.2%) were treated with non-biologic medications that included corticosteroids, thiopurine analogues or 5-ASAs, while 38.8% were treated with biologic medications. On the contrary, most of the Crohn’s disease patients were treated with biologics (72.2%), while 27.8% were treated with corticosteroids or thiopurine analogues.

A total of 28.6% of UC patients were treated with IFX, 6.1% with VDZ, 2% with ADA and 2% with USK. A total of 45.8% of CD patients were treated with IFX, 15.3% with ADA, 8.3% with USK and 2.8% with VDZ (Table 3).

Two patients were lost in the follow-up and there was no recording of their inflammation score after treatment. Those two patients were not included in the study.

### 3.5. IL-21 Expression

#### 3.5.1. A1: IL-21 Expression in IBD Group

There was no significant correlation between IL-21 expression and score reduction in the IBD group. In the biologic-treated group, no difference was found in IL-21 expression before and after treatment. No difference was found between the biologic subgroups either. There was no statistical significance in IL-21 expression compared with the controls (Table 4 and Table 5).

#### 3.5.2. A2: IL-21 Expression in UC and CD Patients

**Figure 1:**
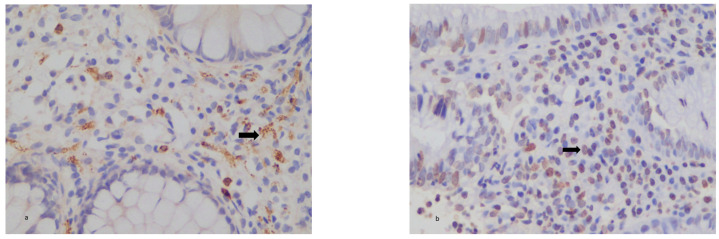
(**a**,**b**) Colonic tissues from UC patient pre- (**a**) and post- (**b**) biologic treatment. Weak (+) tissue staining for IL-21(arrows). ×200.

**Figure 2:**
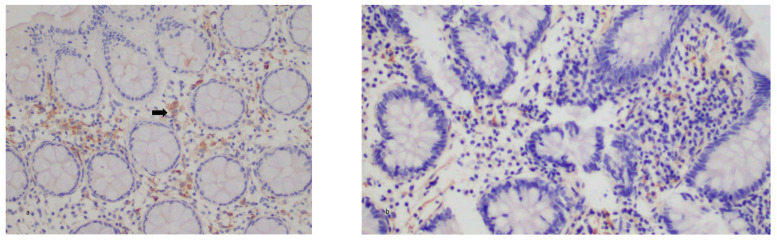
(**a**,**b**) Colonic tissues from CD patient post- (**a**) and pre- (**b**) biologic treatment. Weak (+) tissue staining for IL-21. ×200.

There was no significant correlation found between IL-21 expression and disease activity according to the Mayo activity score or the HBI activity index. There was also no significant correlation between IL-21 expression and the disease phenotype according to the Montreal classification in both the UC and CD groups. There was also no significance in IL-21 expression in the biologic group before or after treatment. There was a statistically significant decrease in the IL-21 expression post-treatment in the VDZ group with UC (*p* < 0.01) but not in the other groups. IL-21 expression was increased in both the UC and CD groups compared with the controls, but no statistical significance was found (Figure 1a,b and Figure 2a,b).

### 3.6. IL-33 Expression

#### 3.6.1. B1: IL-33 Expression in IBD Patients

No correlation was found between IL-33 expression and disease activity according to the HBI score. No correlation was found between IL-33 and the disease phenotype, as per Montreal classification, either (Table 4). A significant increase in IL-33 expression was found in the IBD patient group compared with the controls (*p* < 0.05). In the biologics group, there was a significant increase in IL-33 expression post-treatment (*p* < 0.05). In the subgroup treated with USK, there was a significant correlation of IL-33 expression post-treatment and score reduction (*p* < 0.05) (Table 4 and Table 5).

#### 3.6.2. B2: IL-33 Expression in UC Patients

**Figure 3:**
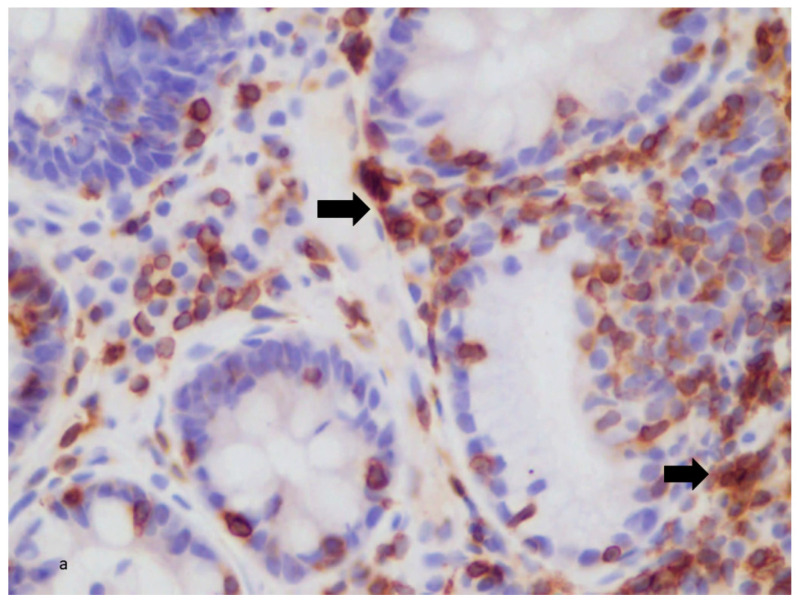

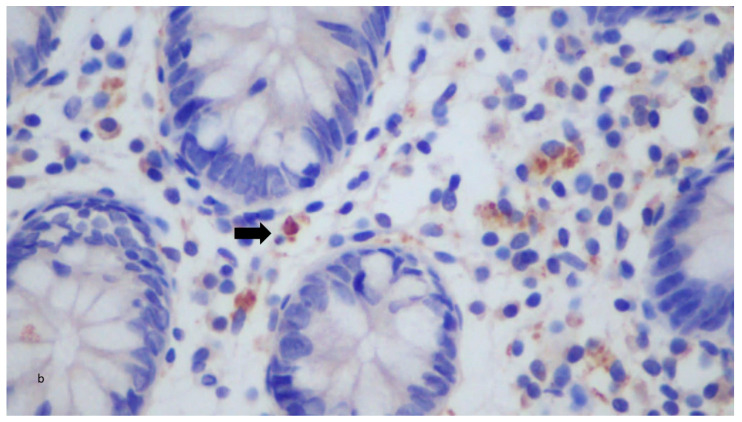
(**a**) Colonic tissue from UC patient post-Infliximab treatment. Strong (+++) tissue staining for IL-33 (arrows). ×400. (**b**) Colonic tissue from UC patient pre-Infliximab treatment. Weak (+) tissue staining for IL-33 (arrows). ×400.

There was a statistically significant increase in IL-33 expression in the UC group (*N* = 49) versus the controls (*p* < 0.05). No significant difference was found in pre-treatment IL-33 expression and disease activity or phenotype. In the biologics group, there was no correlation between the IL-33 expression and the score reduction; however, the post-treatment IL-33 expression was found to be significantly increased (*p* < 0.05). In the biologic subgroups, there was also a significant increase in post-treatment IL-33 expression in the anti-TNFa group (*p* < 0.001) but not in the other subgroups (Figure 3a,b).

#### 3.6.3. B3: IL-33 Expression in CD Patients

A statistically significant increase was found in the IL-33 expression of the CD group compared with controls. There was no correlation between the IL-33 expression and the activity index or the disease phenotype. The post-treatment IL-33 was found to be increased in the biologic-treated group (*p* < 0.05).

**Figure 4:**
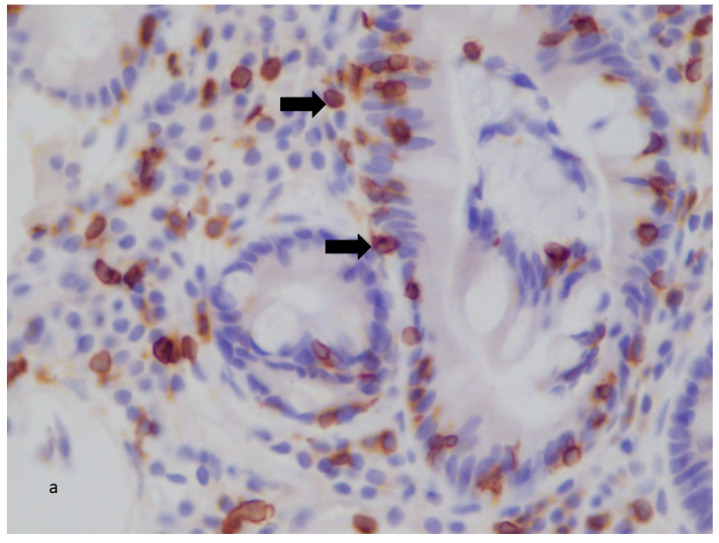

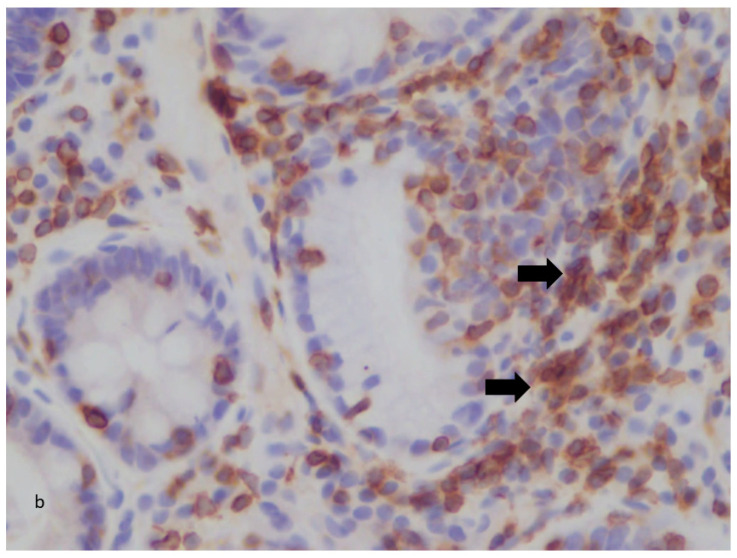
(**a**) Colonic tissue from CD patient pre-Adalimumab treatment. Moderate (++) tissue staining for IL-33 (arrows). ×400. (**b**). Colonic tissue from CD patient post-Adalimumab treatment. Strong (+++) tissue staining for IL-33 (arrows). ×400.

In the biologic subgroups, there was a significant increase in post-treatment IL-33 expression in all the subgroups (*p* < 0.01) (Figure 4a,b).

### 3.7. Intergroup Correlation in Interleukin Expression

A statistically significant positive correlation was found between pre-treatment IL-19 expression, IL-24 and IL-33 in both groups (*p* < 0.01). In the IBD group, there was a significant increase in post-treatment IL-33 and IL-24 (*p* < 0.05).

## 4. Discussion

To our knowledge, there are only limited studies on IBD patients regarding the expression of IL-21 and IL-33 in human intestinal tissue. There were also no data regarding the trends of their expression before and after treatment or their correlation with disease activity post treatment.

Our study has shown an increased expression of IL-21 in the IBD group compared with controls; however, no statistical significance was found (Table 6). There were no statistically significant changes in IL-21 expression after treatment in the UC and CD groups and the biologic subgroups studied. Previous animal studies had contradictory results regarding the role of IL-21 in experimental colitis models, with most of them supporting that IL-21 can ameliorate inflammation [5,16]. On the other hand, the available human studies have shown no significant change in IL-21 expression in IBD patients versus controls, which agrees with our results. In a small study of 44 IBD patients by Liu Z. et al., there was no significant increase in IL-21 in patients with IBD compared with controls with other forms of intestinal inflammation [17].

In another small group of 26 treatment-naive patients, investigating the IL-21 expression before and after IFX administration, there was a significant decrease in IL-21 expression 10 weeks after treatment [18]. Jiang et al., in their study including 40 patients with UC, 20 with CD and 20 healthy controls, identified an increased IL-21 expression in patients with active UC/CD, with significant positive correlation to the clinical disease activity (Truelove and Witts and Harvey-Bradshaw scores for UC and CD, respectively), CRP and PLT levels, endoscopic disease activity and histological activity (*p* < 0.05) [19]. Intestinal IL-21 expression was found to be higher in patients with Crohn’s disease in comparison with UC or healthy controls [20,21,22,23,24]. These were quite small cohorts of patients, using mostly flow cytometry and ELISA and not exclusively IHC. Our results are not in agreement with those studies, and we did not find any significant correlation between IL-21 expression and clinical disease activity or any significant post-treatment reduction in the biologic group/subgroups.

However, we need to mention the limitations of our study at this point, which were the relatively small number of patients included, the single-study method that was used (IHC) and the one-year post-treatment follow-up. The USK group was poorly represented due to funding limitations and the fact that USK had just been introduced in the Greek healthcare system when the data/biopsies were collected. The USK/VDZ subgroups in the UC/CD patient groups were also small to allow definite conclusions on the subgroup analysis. Many patients were also excluded from the study or were lost in follow-up.

Our results (Table 6) strongly support the role of IL-33 as an anti-inflammatory cytokine which helps with mucosal healing and reconstruction. This theory is supported by previous studies with experimental colitis mice models, which have shown that IL-33 receptor ST2 -KO mice had worse colitis, reduced body weight and slower recoveries, with reduced goblet cell populations, and that recombinant IL-33 administration improved their disease activity index [25,26,27,28,29]. The relationship between IL-33 and Th1/Th2/Th9/Treg responses has been poorly defined in human IBD so far, with only limited studies showing an increased IL-33 expression in active IBD cases [30]. Sedhom et al. showed an increased expression of IL-33 in active IBD versus non-IBD [31]. This is in agreement with our results, which showed an increased IL-33 expression in IBD patients compared with controls (Table 6). Seo et al. studied 69 patients with Crohn’s disease, 75 with UC, 45 healthy controls and 74 patients with intestinal bowel disease. A significantly raised IL-33 expression was found in the active UC group versus the Crohn’s disease or intestinal bowel disease groups [28]. More studies, including Kobori et al. and Sun et al., have found increased IL-33 expression in patients with active UC versus active or inactive Crohn’s disease using immunohistochemistry and RT-PCR (*p* < 0.05) [32]. A study by Tahaghoghi-Hajghorbani et al., investigated IL-33’s expression in correlation with disease severity and patients’ age in UC patients and found a lower expression compared with healthy subjects, which was negatively correlated with disease severity [33]. Sponheim et al. also spotted an increased expression of IL-33 in active UC patients vs. patients in remission or healthy controls [34]. Seidelin et al., also found increased IL-33 in active UC patients in their cohort [35]. Wadell et al. demonstrated an increased IL-33 expression in both pediatric and adult UC populations in comparison with controls (*p* < 0.05) in their study [36]. Pastorelli et al. demonstrated similar results via immunohistochemistry and RT-PCR, in patients with active UC. IL-33, and its receptor, ST2, was upregulated in inflamed colonic mucosa (*p* < 0.01) versus controls, with a significant correlation with disease activity in UC (*p* < 0.01). The serum concentrations of IL-33 and ST2 were also found to be upregulated in active IBD patients versus healthy controls, suggesting a possible role as a biomarker of active disease (*p* < 0.01). They also measured the IL-33/ST2 serum expression 2 h after infliximab administration, showing an immediate decrease in IL-33 expression (*p* < 0.01) [37,38].

Those studies used relatively small cohorts of patients with IBD and failed to propose whether IL-33 is protective against intestinal inflammation as the experimental colitis models have shown. Our results are not in agreement with these studies, as the post-treatment IL-33 expression was significantly increased in our biologic group/subgroups in CD and in the biologic group/anti-TNFa group only in UC. In our study, we have supported that IL-33 is protective in both UC and CD subtypes. To our knowledge, this is the first study supporting a likely protective role of IL-33 in CD patients, with a direct relation with the disease activity (Table 6).

Although further research is needed to delineate the exact role of these interleukins in intestinal inflammation, a clear protective role of IL-33 was shown in this study. This cytokine could potentially be used in a prognostic panel model to predict the disease response to treatment.

## Figures and Tables

**Table 2:** Montreal Classification of IBD patients.

Disease	Classification	Frequency	Percent
UC	S1	16	32.7
	S2	15	30.6
	S3	18	36.7
	Total	49	100.0
	E1	5	10.2
	E2	24	49.0
	E3	20	40.8
	Total	49	100.0
CD	A1	1	1.4
	A2	33	45.8
	A3	38	52.8
	Total	72	100.0
	L1	26	36.1
	L2	14	19.4
	L3	30	41.7
	L3 + L4	2	2.8
	Total	72	100.0
	B1	47	65.3
	B2	19	26.4
	B3	6	8.3
	Total	72	100.0

**Table 3:** Treatment choices of IBD patients.

Type of Disease	Type of Treatment	Frequency	Percentage	Valid Percent
UC	ADA	1	2.0	5.3
	IFX	14	28.6	73.7
	USK	1	2.0	5.3
	VDZ	3	6.1	15.8
	Total	19	38.8	100.0
	Other	30	61.2	
	Total	49	100.0	
CD	ADA	11	15.3	21.2
	IFX	33	45.8	63.5
	USK	6	8.3	11.5
	VDZ	2	2.8	3.8
	Total	52	72.2	100.0
	Other	20	27.8	
	Total	72	100.0	

**Table 4:** Pre-treatment IL-21 and IL-33 expression in IBD patients and controls.

Pre-Treatment	IL21			IL33	
Patient Type	Stain Intensity	Frequency	Percent	Frequency	Percent
IBD	0	78	64.5	1	0.8
	+	39	32.3	13	10.7
	++	4	3.3	69	57
	+++	0	0.0	38	31.4
Total	-	121	100.0	121	100.0
Controls	IL21			IL33	
	0	17	85.0	8	40.0
	+	3	15.0	4	20.0
	++	0	0.0	2	10.0
	+++	0	0.0	6	30.0
Total	-	20	100	20	100

**Table 5:** Post-treatment IL-21 and IL-33 expression in IBD patients.

Post-Treatment	IL-21				IL-33	
Type of Treatment	Type of Biologic	Stain Intensity	Frequency	Percent	Frequency	Percent
Biologics	Anti-TNFa	0	57	80.3	0	0.0
		+	12	16.9	5	7.0
		++	1	1.4	22	31.0
		+++	1	1.4	44	62.0
	Total-Anti-TNFa		71	100.0	71	100.0
	USK	0	5	71.4	0	0.0
		+	2	28.6	1	14.3
		++	0	0.0	2	28.6
		+++	0	0.0	4	57.1
	Total-USK		7	9.9	7	100.0
	VDZ	0	3	60.0	0	0.0
		+	2	40.0	0	0.0
		++	0	0.0	3	60.0
		+++	0	0.0	2	40.0
	Total_VDZ		5	100.0	5	100.0
	Total-Biologics		71	-	71	-
Non-Biologic Treatment		0	37	74.0	1	2
		+	13	26.0	1	2
		++	0	0.0	25	50.0
		+++	0	0.0	23	46.0
	Total-Non-Biologics		50	100.0	50	100.0

**Table 6:** Synopsis of study results.

Disease	Method	NR of Patients	IL-21	IL-33
IBD	IHC	121	No correlation was found between IL-21 and disease activity or phenotype.	1. Significant increase in IL-33 expression in IBD patients vs. controls (*p* < 0.05).
				2. Significant increase in post-treatment IL-33 in the biologic group (*p* < 0.05).
				3.Significant correlation between post-treatment IL-33 and score reduction in the USK-treated subgroup (*p* < 0.05).
UC	IHC	49	An increased IL-21 expression was found in UC group vs. controls but was not statistically significant.	1. Significant increase in IL-33 expression in UC group vs. controls (*p* < 0.05).
				2. Significant increase in post-treatment IL-33 expression in the biologics group (*p* < 0.05).
				3.Significant increase in post-treatment IL-33 expression in the anti-TNFa group (*p* < 0.001).
CD	IHC	72	An increased IL-21 expression was found in CD group vs. controls but was not statistically significant.	1. Significant increase in IL-33 expression in CD group vs. controls (*p* < 0.05).
				2. Significant increase in post-treatment IL-33 expression in the biologics group/subgroups (*p* < 0.05).

## Data Availability

The data presented in this study are available on request from the corresponding author. The data are not publicly available due to privacy restrictions.
